# Exogenous selenium mitigates cadmium uptake and accumulation in two rice (*Oryza sativa* L.) varieties in cadmium-contaminated soil

**DOI:** 10.1038/s41598-024-72113-8

**Published:** 2024-09-11

**Authors:** Wenjiang Wu, Deqiang Qi, Yalong Chen, Jiaqi Wang, Ganggang Zhang, Qinghua Wang, Hongbin Niu, Quanzhi Zhao, Ting Peng

**Affiliations:** 1 Innovation Center of Henan Grain Crops, Henan Key Laboratory of Rice Biology, Henan Agricultural University, Zhengzhou, 450046 Henan People’s Republic of China; 2 College of Agronomy, Henan Agricultural University, Zhengzhou, 450046 Henan People’s Republic of China; 3 College of Horticulture, Henan Agricultural University, Zhengzhou, 450046 Henan People’s Republic of China; 4 Forestry and Fruit Research Institute of Beijing Academy of Agricultural Sciences, Beijing, 100089 People’s Republic of China; 5 College of Agronomy, Guizhou University, Guiyang, 550025 Guizhou People’s Republic of China

**Keywords:** Cadmium, Ratoon rice (RR), Selenium, Se-enriched, Translocation factor, Environmental chemistry, Plant physiology

## Abstract

Rice grown in cadmium (Cd)-contaminated soil, is a potential threat to human health, but exogenous selenium (Se) application on rice can mitigate Cd toxicity. However, the mechanisms underlying Se mitigation of Cd stress in ratoon rice (RR) are still poorly understood. We conducted a pot experiment with moderate Cd-contaminated yellow–brown paddy soil on two rice varieties ‘Taoyouxiangzhan’ (TX) and ‘Liangyou 6326’(LY). For all treatments, 1.0 mg kg^−1^ sodium selenite solution was added to soil. Treatment T1 was sodium selenite only, and in the other treatments 100 mg L^−1^ Se solution was sprayed on the leaves at seedling stage (T2), at tillering stage (T3), and in early anthesis stage (T4). Se treatments decreased Cd accumulation in rice grains and herbage. Under foliar spraying 100 mg L^−1^ Se at the seedling + 1.0 mg kg^−1^ Se in soil (T2), leaf Cd content decreased 16.95% in the current season and grains content decreased 46.67% in the subsequent season. Furthermore, grain Se content increased 0.94 mg kg^−1^ for the TX variety combined with the analysis of Cd bio-accumulation factor in grains, and Se treatments effectively decreased Cd grain concentrations due to reduced Cd translocation from roots to grains. TX variety rice showed a more pronounced response to Se treatments than LY.

## Introduction

Environmental pollution has become one of the main challenges for human health. Cadmium (Cd) as one of the highly toxic pollutants, is deleterious for biological systems through its uptake and accumulation in phototrophs and consequent trophic transportation, causing serious environmental problems worldwide^1–3^. Cd is ranked seventh in the top 20 toxic metals list and classified as a group 1 carcinogen^3^. Rice (*Oryza sativa* L.) is one of the most important global food crops, and China is the world's largest rice producer and consumer, accounting for nearly 30% of global rice production and 28% of global rice consumption^4^. In Asia, rice grains are a staple food for about two billion people with 20–40 µg Cd intake daily^5^. Cd accumulation in rice plants contaminates the food chain, disrupts human metabolism and causes various health problems^1,6^. Thus, the high-concentration Cd in paddy soils is a global environmental issue, and Cd translocation and subsequent accumulation in rice grains has attracted global attention^2,7^.

Increasingly, it appears that Cd translocation from soil to rice roots may be reduced by controlling soil pH and redox potential^8^. Soil pH is the key factor that regulates the amount of exchangeable soil Cd for plant uptake, and acidic soils promote Cd availability for plants^9^. With a decrease in soil pH, Cd is transformed from fixed to readily mobile form so that soil Cd availability is increased, leading to potential nutritional toxicity for plants^10^.

Actually, the right selenium (Se) application in rice can decrease Cd uptake and toxicity in Cd-contaminated soils^7,11,12^. Se as a trace element, is an indispensable element for human and animal health, but may adversely affect human health when in excess or deficient^13–15^. It is well known that Se application is a cost-effective strategy, and significantly reduces plant root and shoot Cd concentrations^14,16–21^. Recently, Feng et al.^22^ proposed four possible mechanisms for how Se influences uptake and translocation of metal(loid)s in plants: (1) targeting of the physicochemical processes in soils by impacting metal(loid)s bioavailability; (2) associated with plant root morphology regulation; (3) relies on stimulation of increased metal(loid)s sequestration by Se in root iron plaques, cell walls, or vacuoles; and (4) regulation of uptake and transport pathways. Therefore, Se may have wide application prospects, because it can not only relieve stress resulting from heavy metal toxicity, such as Cd, but also satisfy the daily requirement for human Se intake^2^. Se is an essential element for animal and humans, but some populations around the world are suffering from Se deficiency, in particular in China, because of unbalanced Se distribution^23^. Administering exogenous Se of increasing concentration via food crops is an effective way to meet human demand in Se-deficient areas^2,24^. Conversely, too high Se concentrations in the edible parts of rice may be toxic, posing a public health risk^13,25^. Therefore, exploring the Se application range in rice is necessary to reduce Cd accumulation, due to a narrow safety threshold. Based on Se-Cd elemental antagonism, this study focused on mitigating Cd accumulation in rice by increasing Se content. Development of ratoon rice (RR) is one method to increase food production with minimal agricultural inputs^26,27^.

RR is characterized by its high grain quality, which is mainly associated with lowered temperature after the heading stage^14,28^. Rice grain quality improvement has become popular in agronomy, and plays an increasingly important role in determining rice consumption^14,26,29^. In the agricultural context of labor shortage, water scarcity and increasing production costs, the planting scale of RR is a promising system to ensure more development^30^. In particular, because of the forage shortage for animal husbandry development and land competition for grain production, the recent promotion and application of grain and feed dual-purpose crops is important for coordinated grain and forage production. Therefore, we adopted a planting pattern of current-season rice as herbage and a subsequent rice season for grain. However, there have been no reports of research on Se enrichment and Cd reduction in RR.

Hence, the objective was to assess the environmental impacts of RR production and to identify RR production hotspots. The two rice varieties growing in Cd-polluted paddy soil were investigated for Cd content in different tissues after four Se treatments during two seasons, to explore scientific approaches to minimize Cd in rice grains and potential Cd-translocation mechanisms. Based on a framework of RR and Se/Cd relationship-oriented expressions, this study is the first to comprehensively increase Se and reduce Cd in RR production in central and southern China. The results will provide a basis for growers to implement RR in China that will increase productivity and environmental sustainability.

## Materials and methods

### Plant materials

#### Rice varieties

‘Liangyou 6326’ (LY) and ‘Taoyouxiangzhan’ (TX) hybrid rice varieties bred by Chinese researchers were used in the experiment. LY (National certification No. 2007013) variety belongs to the two-line hybrid rice of the indica type (Taonong1A × Huanghuazhan). It has 123 days average growth period, grows to 110.3 cm tall, with strong stems and growth trend. The leaves are dark green, straight and sword-shaped. The color changes well during maturity. The average yield from 2020 to 2021 was 554.21 kg/666.7 m^2^. TX (National certification No. 20210307) variety belongs to the indica type three line hybrid rice of Xuan 69S × WH26. Its entire reproductive period is 113 days, and the plant grows to 100.8 cm high, with vigorous growth, strong tillering ability, good color setting in the later stage, and moderately low temperature tolerance. The average yield from 2021 to 2022 was 543.19 kg/666.7 m^2^. These two varieties are suitable for rice cultivation in Henan Province, China. Seeds of the two tested varieties were obtained from the Collaborative Innovation Center of Henan Grain Crops, Henan Agricultural University. Initial seed moisture content on a dry weight basis was 10.9 and 10.6% for LY and TX, respectively. These two varieties are used for dual-purpose RR with year-round and excellent forage grains. All the plant experiments complied with the IUCN Policy Statement on Research Involving Species at Risk of Extinction and the Convention on the Trade in Endangered Species of Wild Fauna and Flora.

#### Soil characteristics

##### Soil sample preparation

Fresh paddy soil (yellow–brown soil) was collected from a rice field within a 0–20 cm depth soil cultivation layer using the five-points method in Lilou Village, Guangshan County, Henan Province (112°58ʹ–113°30ʹ N, 27°54ʹ–28°22ʹ E). The soil contained 38.3 g kg^−1^ of organic matter, 2.97 g kg^−1^ of N, 7.2 mg kg^−1^ of available phosphorus, 153 mg kg^−1^ of rapidly available potassium, 1.33 mg∙kg^−1^ of Cd, and 0.43 mg kg^−1^ of Se, and soil pH was 5.8. Cadmium pollution in the paddy field mainly arises from sewage from nearby chemical plants. The paddy soil belonged to the moderate Cd-contaminated soil, according to Soil Environmental Quality Risk Control Standard for Soil Contamination of Agricultural Land (GB 15618-2018, China).

##### Pot preparation and experimental layout

Large plastic (PVC) pots (25-cm diameter and 20-cm height) were used for greenhouse preparations. Based on conventional fertilization practices for rice production, the basal fertilizer was composed of 150 mg kg^−1^ N, 100 mg kg^−1^ P_2_O_5_, and 100 mg kg^−1^ K_2_O. After thorough mixing, 5 kg soil was put in each plastic pot and three rice seedlings planted. There were 5 pots per treatment, for a total number of 60. Tap water (Cd and Se concentration were BDL) was used for irrigation in the entire experiment. Irrigation was controlled so that a constant 3–4 cm of standing water was maintained during the growth period and irrigation was stopped ten days before rice grain harvest. The entire experiment was arranged in a complete randomized design (CRD) with thirty (30) treatment combinations replicated thrice. Two different rice varieties with contrasting grain morphology, and four Se treatments were designed as follows:

- CK, 0 mg kg^−1^ Se in soil + foliage spraying water;
- T1, 1.0 mg kg^−1^ Se in soil + foliage spraying water;
- T2, 1.0 mg kg^−1^ Se in soil + foliage spraying 100 mg L^−1^ Se once at the seedling stage;
- T3, 1.0 mg kg^−1^ Se in soil + foliage spraying 100 mg L^−1^ Se once at the seedling and once at tillering stages;
- T4, 1.0 mg kg^−1^ Se in soil + foliage spraying 100 mg L^−1^ Se three times (seedling stage, tillering, and early anthesis stages).

Sodium selenite (analytical pure grade, Nanjing Chemical Reagent Co., Ltd, China) was applied in soil and selenium sprayed. For soil application, the soil was put on a plastic cloth, and 1.0 mg kg^−1^ Se evenly sprayed on it. At the same time, base fertilizer of N-150 mg kg^−1^, P_2_O_5_-100 mg kg^−1^, and K_2_O-100 mg kg^−1^, was mixed well and put into the pot. After 3 days of water logging balance, the first season rice seedlings were transplanted (3 per pot). The current-season rice was clipped at 15 days after T4 treatment, on 30 June, 2021, and the remaining stubble height was maintained at 25 cm for the subsequent season. Appropriate amounts of nitrogen, phosphorus, and potassium fertilizers were used in the subsequent rice season. Water management of both the previous and subsequent rice seasons was 5 cm flooding, and when there was no clear water in the topsoil of the pot, water was replenished to 5 cm flooding. Disease, pest, and weed management was performed in accordance with the local field rice production.

The same management protocols were applied in the subsequent rice season, which was harvested on 21 October, 2021. The samples (including grains, roots, stems, and leaves) were cleaned with distilled water. The roots were extracted with Dithionite-Citrate-Bicarbonate (DCB) solution to determine Cd and Se contents. All samples were dried in an oven at 65◦C to constant weight, and ground into powder for further analysis.

#### Sample collection and determination

Using the five-points method, 200 g soil samples were taken from each pot at 0–20 cm depth, air dried, and sieved (20-mesh sieve) to determine soil pH and available Cd. Soil pH was measured with deionized water (1:2.5 soil to water ratio) using a pHs-3C meter (Leici, Shanghai, China). Available Cd was determined using the 0.01 M∙L^−1^ CaCl_2_ method. The soil/water (1:5) was shaken at 160 r∙min^−1^ for 2 h at 25 °C, and filtered with a quantitative filter paper. Cd content of the filtered samples was determined by inductively coupled plasma mass spectrometry (Agilent ICP-OES 5110, USA).

For Cd content in various rice tissues, 0.20 g samples were digested with a HNO_3_:H_2_O_2_ (8:1) solution, transferred into a 25 mL colorimeter tube, and filtered with a quantitative filter paper. Cd content of the filtered samples was determined by inductively coupled plasma mass spectrometry (ICP-MS, Varian, USA). For Se content in various rice tissues, 0.50 g samples were digested with 10 mL HNO_3_:HCLO_4_ (4:1), transferred into a 25 mL colorimeter tube, and filtered with a quantitative filter paper. Se content of the filtered samples was determined with an atomic fluorescence photometer (AFS-830, Shanghai Shuangxu Electronics Co., Ltd., China).

Green straw from current-season rice was clipped as silage. After air drying, its quality was measured, and the first season dry forage yield calculated. Three representative pots were selected for each treatment to calculate grass yield. Meanwhile, some quality traits were evaluated. Crude protein content was measured using the GB/T 6432-2018 method, and crude fiber content determined by filtration according to GB/T 6434-2006. Crude ash and starch contents were determined according to GB/T 6438-2007 and GB/T 20194-2018, respectively. Six pots were randomly selected for each treatment. All procedures were carried out in accordance with relevant guidelines.

#### Translocation factor (TF) and bioconcentration factor (BCF)

We calculated translocation factors (TFs) and bioconcentration factors (BCFs) using the following formulas^31,32^.

$$ {\text{TFroot-straw }} = {\text{ Cd concentration in straw/Cd concentration in roots}}; $$

$$ {\text{TFstraw-brown rice }} = {\text{ Cd concentration in brown rice/Cd concentration in straw}}; $$

$$ {\text{TFstraw-rice husk }} = {\text{ Cd concentration in rice husk/Cd concentration in straw}}; $$

$$ {\text{BCF = Cd concentration inplantpartt/Cd concentration in soil}}. $$

#### Statistical analysis

Data was expressed as a mean value ± standard error (n = 3). Statistical analysis was performed using SPSS 22.0 software. ANOVA was used to evaluate significant differences among treatments using Duncan's new multiple range test (SSR) (P < 0.05 and P < 0.01) and multiple comparison analysis. Pearson’s correlation and nonlinear regression analysis were performed to verify correlations between tissue Se and Cd concentrations. Figures were drawn using Microsoft Excel 2010. Each treatment was replicated thrice.

## Results

### Effect of Se treatments on Cd content in different rice tissues

**Fig. 1:**
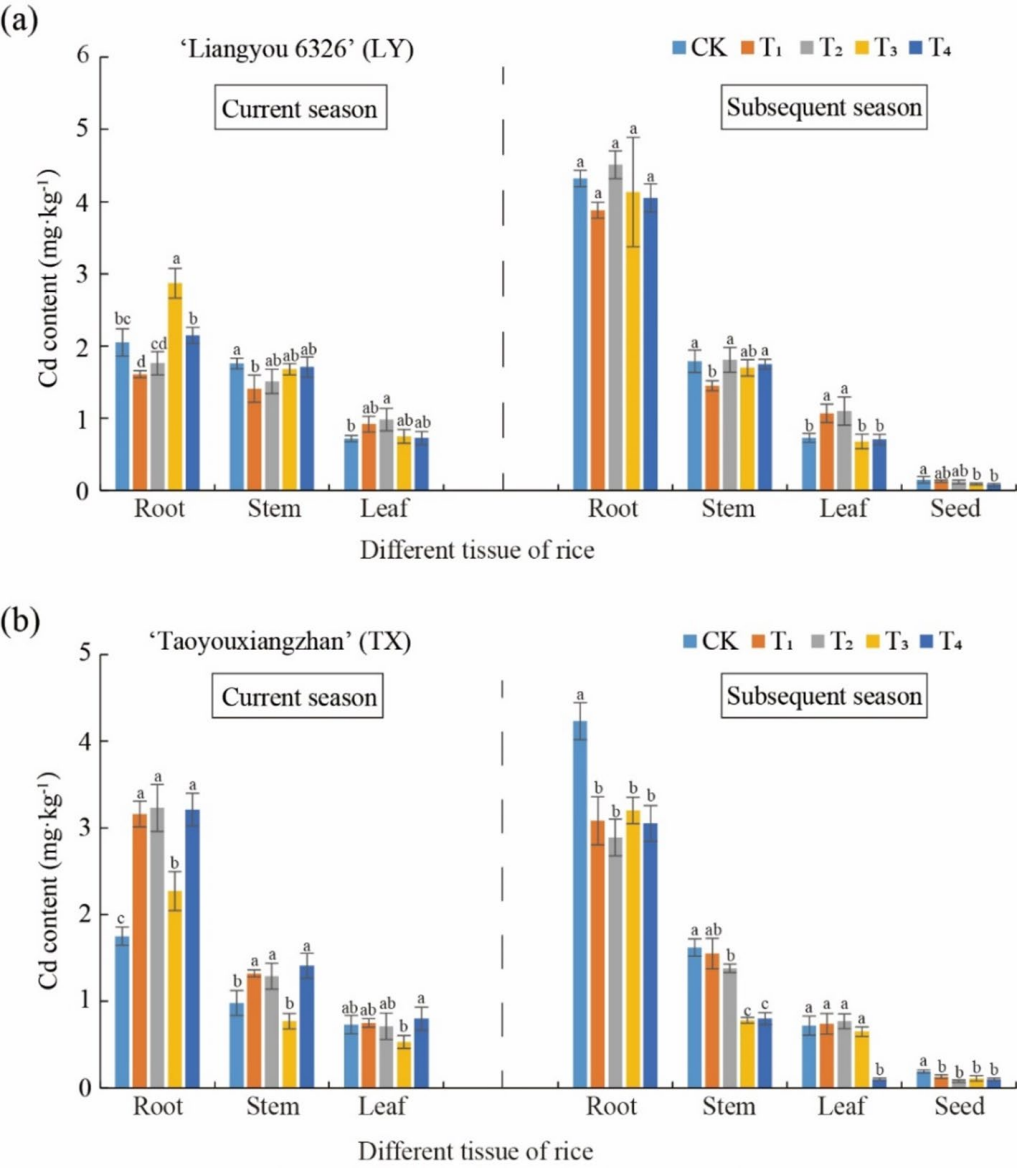
Cd content in different tissues of LY (a) and TX (b) ratooning rice after Se treatments. RR ratooning rice. The treatments were set as follows. CK: 0 mg kg^−1^ sodium selenite (Se) in soil + foliage spraying water;T1: 1.0 mg kg^−1^ Se in soil + foliage spraying water; T2: 1.0 mg kg^−1^ Se in soil + foliage spraying 100 mg L^−1^ Se once at the seedling stage; T3: 1.0 mg kg^−1^ Se in soil + foliage spraying 100 mg L^−1^ Se once at the seedling and once at tillering stages; T4: 1.0 mg kg^−1^ Se in soil + foliage spraying 100 mg L^−1^ Se three times (seedling stage, tillering, and early anthesis stages). The same below (Figs. 2, 3). The lowercase letters in the table represent significant differences at the 0.05 level, the same letters represent insignificant differences, and different letters represent significant differences. Data are means ± SD (n = 3).

Cd content among rice tissues was in the order roots > stems > leaves > grains between LY and TX, indicating that rice absorbed Cd and up-transported it mainly through roots (Fig. 1). Compared with the current season of LY rice, Cd accumulated significantly in roots in the subsequent season (Fig. 1a). However, the effect of Se treatments on tissue Cd content displayed diverse changes. For example, in the T1 treatment, root and stem Cd contents were significantly lower than in CK. Meanwhile, in T1, T2, and T4, leaf Cd content increased, whereas T3 had reduced Cd content (Fig. 1a). In the subsequent LY rice season, Cd content in roots did not significantly differ among treatments. Notably, in T1 stem Cd content decreased significantly, compared with CK. Moreover, in T3 and T4 treatments grain Cd contents decreased. Se treatments decreased grain Cd content by 20% (T3; Fig. 1a), which was lower than 0.2 mg kg^−1^ Cd (National Standard of Cd)^33^. For the current TX rice season, the four Se treatments increased root and stem Cd content significantly more than in leaves. Conversely, foliar spraying twice significantly reduced Cd content in both stems and leaves by 14.20 and 16.95% (P < 0.05), respectively. The four Se treatments lowered root and stem Cd content in the subsequent season, especially in grains with a 46.67% decrease (T2, i.e. spraying Se solution once on the leaves after adding Se fertilizer to the soil), which was the optimal treatment (Fig. 1b). In the T3 and T4 treatments, spraying Se solution two and 3 times on the leaves after adding Se fertilizer to the soil, significantly reduced grain Cd content, compared to the control by 17.5 and 20.01%, respectively (Fig. 1b).

### Effect of Se treatments on Se content in different rice tissues

**Fig. 2:**
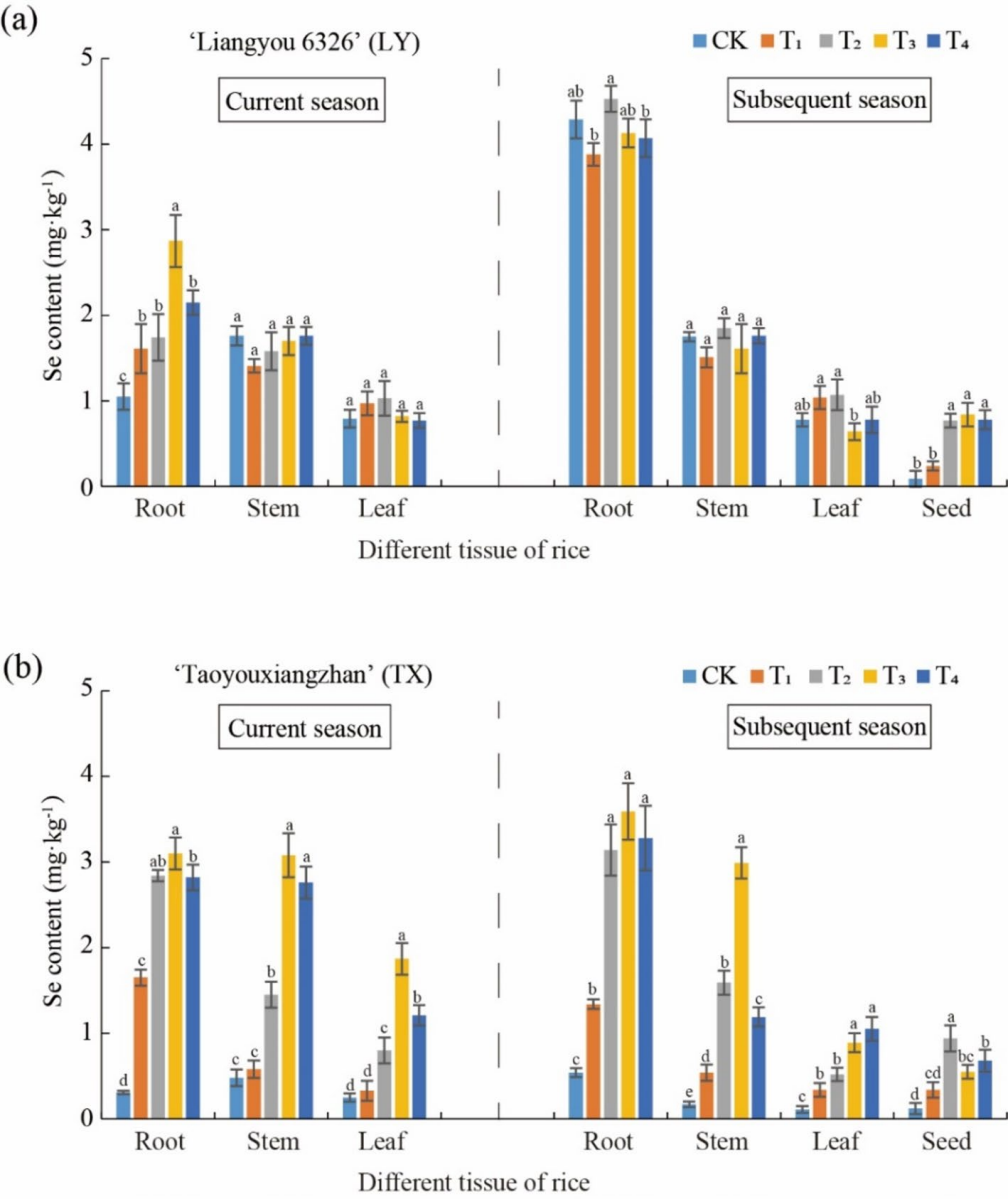
Se content in different tissues of LY (**a**) and TX (**b**) ratooning rice after Se treatments.

As shown in Fig. 2a, after Se treatments, Se accumulated significantly in roots in the current LY rice season, for example, Se content in the T3 treatment was 2.87 mg kg^−1^, and compared to the control increased 2.73 fold (Fig. 2a). However, there was no significant difference in stems and leaves (P < 0.05). Importantly, grain Se content increased after Se treatments, especially after the three Se foliage spraying treatments (Fig. 2a). Rice grain Se content significantly increased in the subsequent season after the Se foliage spraying treatments (0.24, 0.77, 0.84, and 0.78 mg kg^−1^, respectively), compared to 0.09 mg kg^−1^ in the control, which was up to 9.33 fold higher than CK.

For TX rice, Se content significantly increased in the current season or subsequent season after Se foliage spraying treatments, compared with the control group. Se content of each Se treatment combination significantly increased 2.85 fold in roots, 3.76 fold in stems and 2.34 fold in leaves more than CK (Fig. 2b). Compared to the control treatment, the increase in rice grain Se content was slightly lower (0.94 mg kg^−1^), but the difference was significant. Se as a trace element, is essential and beneficial for human health. In the present study, Se content was from 0.09 to 0.94 mg kg^−1^ in LY and TX rice grains, lower than 1.0 mg kg^−1^ which is the standard of Hunan Selenium Content Requirements of Selenium-rich Agricultural Products (T/HNFX 001-2017, China)^33^. The above results showed that 1.0 mg kg^−1^ Se amendment in soil could both reduce Cd stress and increase Se content in Cd-contaminated soil.

### Effect of Se treatments on the Cd translocation factor (TF)

**Fig. 3:**
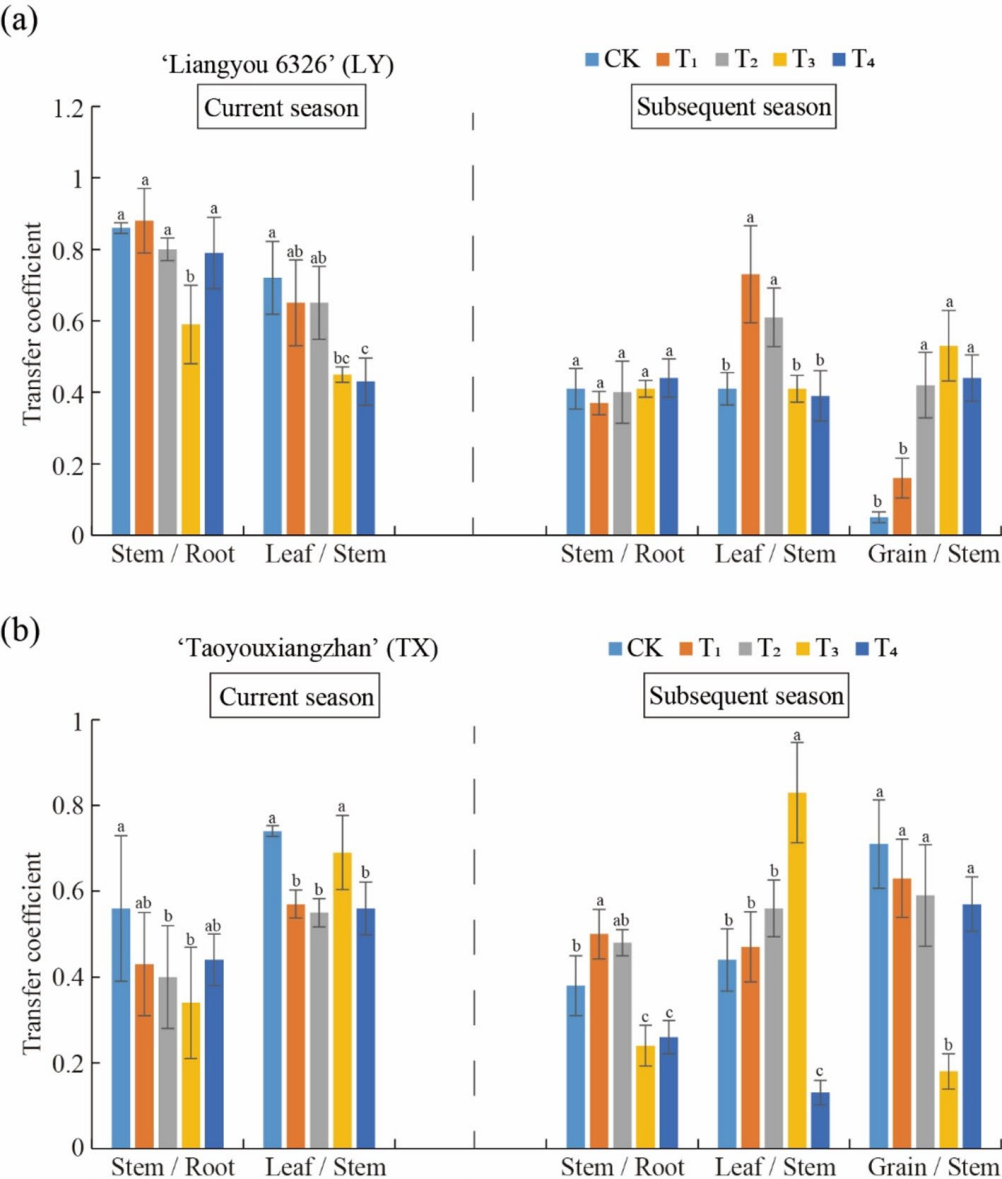
Effect of Se treatments on Cd transport coefficient in ratnoon rice tissues.

The translocation factor (TF) was used to analyze the effect of Se treatments on rice Cd translocation. TFs in LY and TX after Se treatments decreased in the current season, but most increased in the subsequent season (Fig. 3). Moreover, compared with CK,in the subsequent LY season, grain/stem TF of T2, T3, and T4 treatments significant increased, while a similar result was obtained for leaf/stem TF of T1 and T2 treatments (Fig. 3a). For the current TX season, T2 significantly decreased stem/root TF by 28.57%, and leaf/stem TF by 64.71%, compared with the CK. T3 treatment also significantly increased leaf/stem TF, but lowered both stem/root and grain/stem TF in the subsequent season, compared with the CK (Fig. 3b). Collectively, Se treatments effectively decreased Cd translocation from roots to grains in TX rice. Overall, although Se application in paddy soils reduces Cd, in practice, other environmental factors should also be considered.

### Effect of Se treatments on herbage yield and quality

LY and TX dry herbage and silage yield were highest in T3 treatments, followed by T2 treatment, with a significant difference compared with CK (Table 1). After one and two Se treatments, LY harvest was 519.83 and 522.02 g of dry forage per pot, which was 5.18 and 5.67% higher than the control, respectively. In terms of quality, hay crude protein content increased by 5.40 and 15.85%, respectively. Simultaneously, hay Se content increased by 0.08 and 0.1 mg kg^−1^ compared to the control, and the corresponding Cd decreased by 0.01 and 0.28 mg kg^−1^. TX grass yield was slightly lower than LY, but the effect of treatment was also obvious, and compared with the control, crude protein content increased by 22.63 and 19.88%, respectively. TX hay Se content increased by 0.72 and 1.91 mg kg^−1^ compared to the control, while the corresponding Cd element decreased by 0.12 and 0.25 mg kg^−1^. Differences between the treatment and control of the two varieties were significant. However, the lowest yield appeared in the LY T4 and TX T1 treatments among Se treatments. Se treatments resulted in a significant increase in crude protein content in the two rice varieties, with the greatest effect in LY T3 and TX T1, respectively. Additionally, crude fiber content significantly decreased in the four LY Se treatments, and significantly increased in the TX T4 treatment. In LY and TX, a similar trend in starch content was observed, and significantly higher than their CKs. Also, Se treatments greatly reduced Cd content in the two rice varieties, except in the LY T4 treatment.

**Table 1:** Effects of Se fertilization on the yield and quality of current-season herbage of RR.

Treatment		Dry herbage yield (g pot^−1^)	Silage Yield (kg pot^−1^)	Crude fiber content (g)	Crude protein content (%)	Starch content (%)	Crude ash content (%)	Se content (mg kg^−1^)	Cd content (mg kg^−1^)
LY	CK	494.32 ± 24.06 b	703.16 ± 28.14 b	26.43 ± 1.81 b	8.33 ± 0.76 c	3.15 ± 0.88 d	11.37 ± 0.49 d	1.20 ± 0.15 c	1.25 ± 0.38 b
	T1	493.24 ± 16.28 b	701.88 ± 16.57 b	25.82 ± 2.17 c	8.58 ± 0.81b	3.49 ± 0.29 c	11.39 ± 0.44 d	1.17 ± 0.19 cd	1.14 ± 0.21c
	T2	519.83 ± 13.88 a	739.68 ± 31.05 a	24.53 ± 0.89 c	8.78 ± 0.81 b	3.96 ± 0.24 a	11.86 ± 0.55 c	1.28 ± 0.16 c	0.97 ± 0.28 d
	T3	522.02 ± 13.88 a	743.09 ± 28.32 a	25.81 ± 0.93 c	9.65 ± 0.42 a	3.86 ± 0.52 ab	11.66 ± 0.55 d	1.30 ± 0.23 c	1.24 ± 0.36 b
	T4	468.32 ± 28.32 c	666.25 ± 17.66 c	25.31 ± 0.79 c	8.71 ± 0.45 b	3.44 ± 0.39 c	11.85 ± 0.57 c	1.11 ± 0.34 d	1.31 ± 0.36 a
TX	CK	437.87 ± 27.30 d	622.99 ± 24.13 d	27.43 ± 1.52 b	8.00 ± 0.54 d	3.01 ± 0.38 d	12.08 ± 0.60 b	0.41 ± 0.15 e	1.12 ± 0.20 c
	T1	447.46 ± 27.03 d	636.65 ± 25.54 d	25.87 ± 1.02 c	9.81 ± 0.45 a	3.27 ± 0.39 d	12.11 ± 0.40 b	0.51 ± 0.17 e	0.99 ± 0.17 d
	T2	478.65 ± 27.21 c	681.05 ± 26.42 c	27.30 ± 1.19 b	9.59 ± 0.52 a	3.57 ± 0.42 c	11.75 ± 0.36 cd	1.13 ± 0.19 d	0.87 ± 0.14 e
	T3	492.61 ± 29.6 b	700.97 ± 32.05 b	27.53 ± 1.95 b	9.04 ± 0.86 b	3.71 ± 0.24 b	11.96 ± 0.22 b	2.32 ± 0.52 a	0.83 ± 0.20 e
	T4	461.04 ± 27.82 c	656.0 ± 23.65 c	30.16 ± 0.50 a	8.79 ± 0.44 b	3.49 ± 0.30 c	12.34 ± 0.39 a	1.77 ± 0.68 b	1.06 ± 0.21 d

RR ratooning rice. LY: Liangyou 6326; TX: Taoyouxiangzhan. The treatments were set as follows. CK: 0 mg kg^−1^ sodium selenite (Se) in soil + foliage spraying water;T1: 1.0 mg kg^−1^ Se in soil + foliage spraying water; T2: 1.0 mg kg^−1^ Se in soil + foliage spraying 100 mg L^−1^ Se once at the seedling stage; T3: 1.0 mg kg^−1^ Se in soil + foliage spraying 100 mg L^−1^ Se once at the seedling and once at tillering stages; T4: 1.0 mg kg^−1^ Se in soil + foliage spraying 100 mg L^−1^ Se three times (seedling stage, tillering, and early anthesis stages). The same below (Table 2). The lowercase letters in the table represent significant differences at the 0.05 level, the same letters represent insignificant differences, and different letters represent significant differences. Data are means ± SD (n = 3).

Overall, based on the comprehensive analysis of straw yield, crude protein, selenium, and Cd content, the optimal Se treatment for LY was T3, namely, spraying Se solution twice on the leaf surface, while for TX, T2 and T3 were better Se treatments.

### Effect of Se treatments on soil pH and available Cd content

In the soil planted with TX, pH of each combination changed significantly, initially increasing and then decreasing in the current season, and then decreasing again in the subsequent season. Correspondingly, available soil Cd content increased slightly in the current season, and then decreased and again increased in the subsequent season treatment combination, while pH decreased significantly with increasing treatment time. For the LY variety, Se application in the subsequent season decreased soil pH by an average of 0.12 units compared to the control group, while the average Cd content of the three Se spraying treatments decreased by 9.38% (P < 0.05).

For TX varieties, compared with the control, soil pH in the subsequent season decreased by 0.085 units on average, but available soil Cd content of the Se treatment combinations increased initially, and then decreased. Overall, the effect of Se application on soil pH and available Cd content differed between the two varieties. Compared with the current season, soil pH of the subsequent season decreased by 0.02–0.43 units, and available soil Cd content increased by 7.0 to 10.26%, respectively.

### Correlation analysis of Se content among rice tissues

**Fig. 4:**
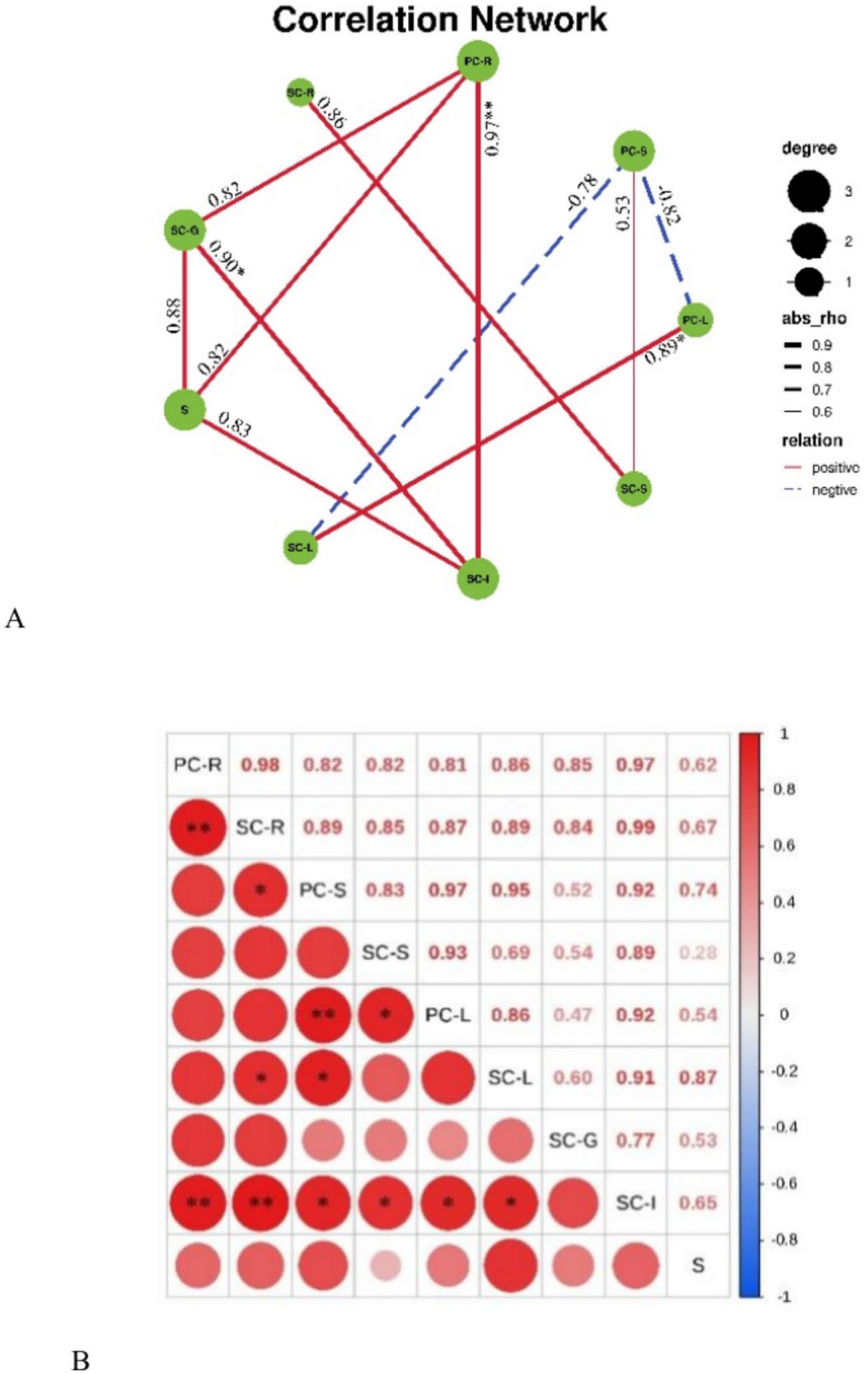
Correlation analysis of selenium content among different tissues in Liangyou (LY) ratooning rice. SC: current season; SS: subsequent season; root; S: stem; L: leaf; G:grain; S: soil. The same below (Fig. 5).

**Fig. 5:**
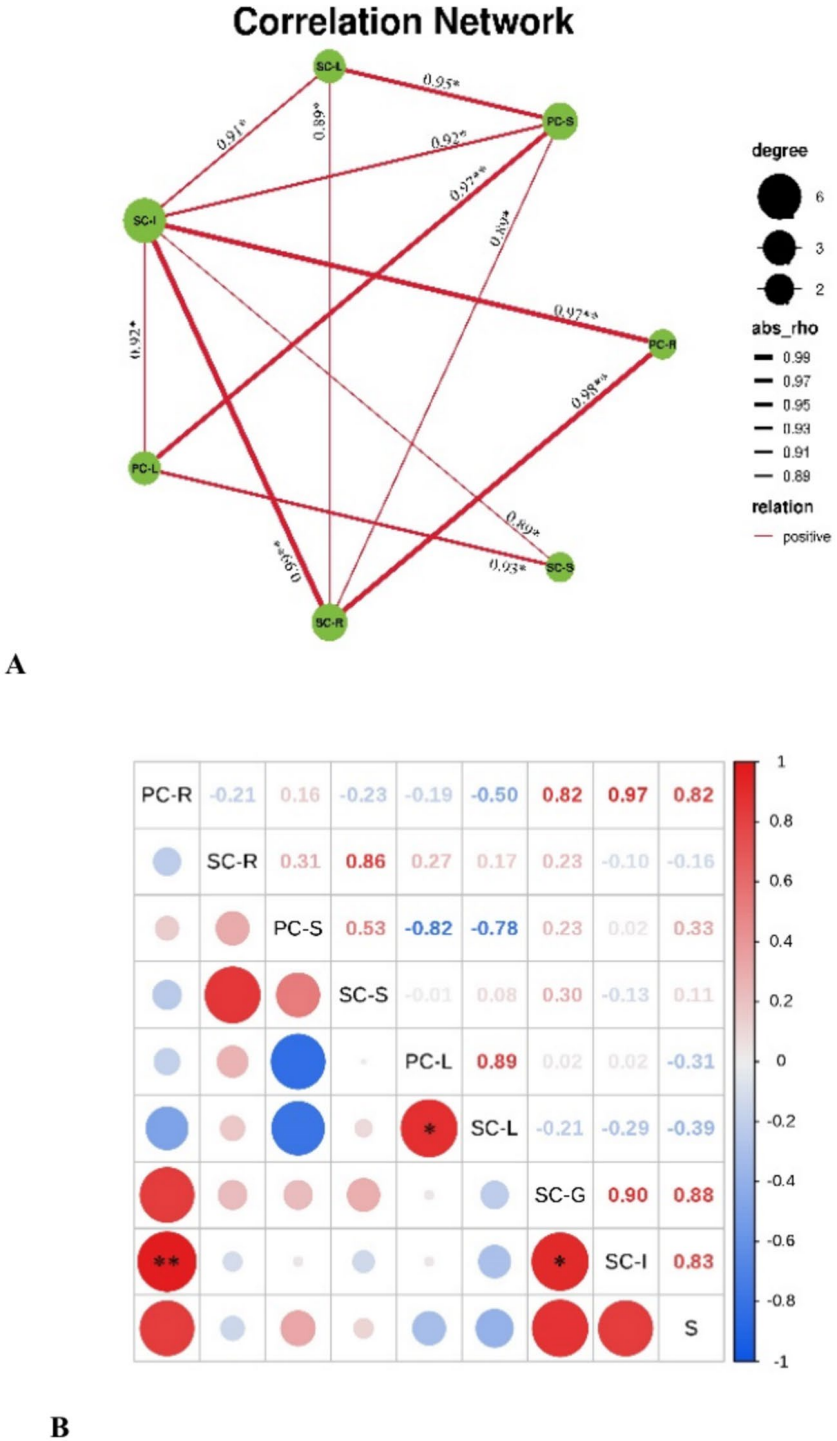
Correlation analysis of Se content among different tissues in Taoxiang (TX) ratooning rice.

The correlation analysis showed obvious difference between the two varieties (Figs. 4, 5). In LY, current season stems were negatively correlated with leaves of the two seasons. A significant correlation between current season leaves and those of the subsequent season indicated that Se accumulation in the subsequent season leaves mainly resulted from current season leaves. Similarly, Se accumulation in grain may be from current season roots and soil (Fig. 4A,B). In TX rice, highly significant positive correlations were observed, e.g., roots in the current season with in those in the subsequent season, stems with leaves in the current season, and grains with roots in both seasons, suggesting that the Se in grains was mostly translocated by roots (Fig. 5A,B).

### Cd bio-accumulation factor analysis in grains

**Fig. 6:**
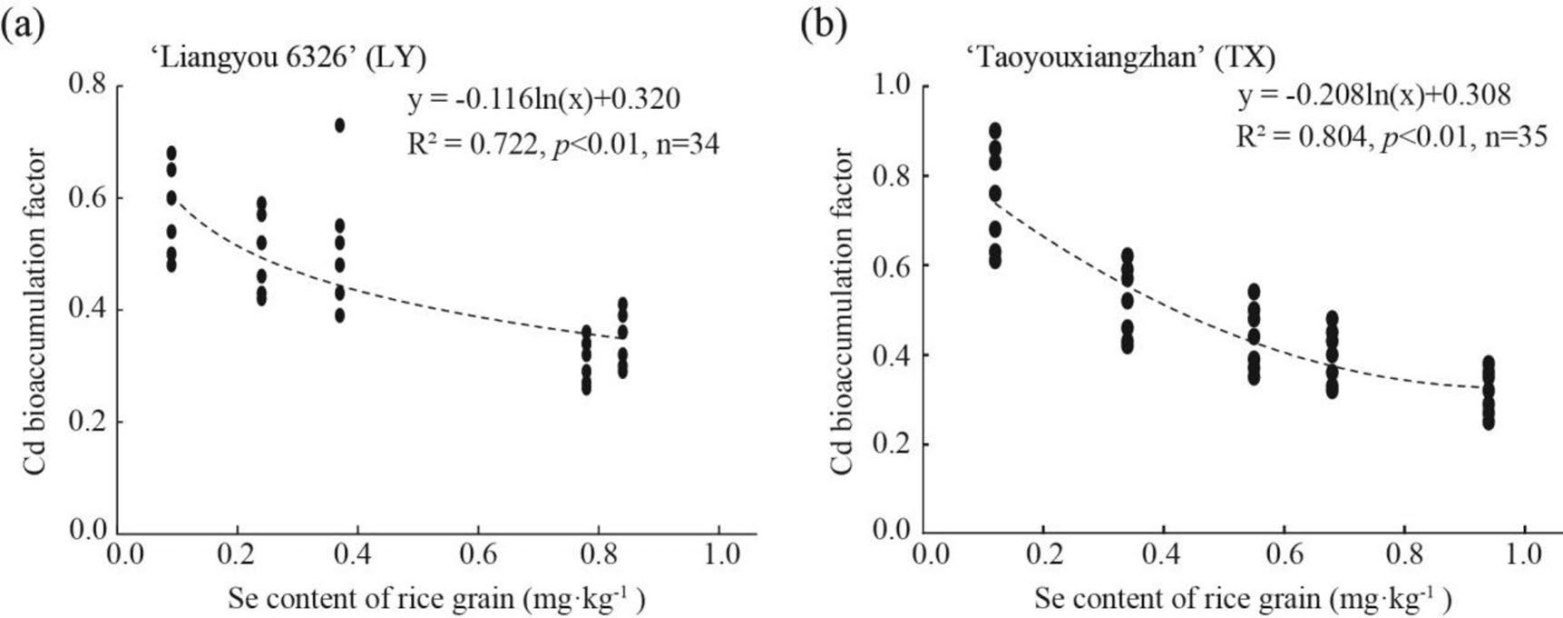
Relationship of Cd bio-accumulation factor with Se concentration in ratooning rice.

To further explore the ability of grains to uptake Cd from soil after Se addition, the Cd bio-accumulation factor in rice grains was investigated. Cd bio-accumulation factor and Se content were negatively correlated in LY (-0.853 Pearson coefficient, P < 0.01, n = 35), and TX (-0.864 Pearson coefficient, P < 0.01, n = 34) (Fig. 6). Moreover, Cd enrichment factor in grains decreased (natural logarithm) with increasing Se and more quickly in TX than in LY (Fig. 6), indicating that Se accumulation in grains could reduce Cd accumulation by improving Se uptake, with a stronger effect in TX.

## Discussion

The effect of Se application of different concentrations on Cd uptake and transport in RR fields with moderate Cd pollution was explored. Cd content in different RR variety tissues was in the order root > stem > leaf > grain (Fig. 1), which was consistent with previous rice research^34,35^, indicating that Cd in rice plants came mainly from the soil and was transferred upwards from the root.

The maximum Cd concentration in soil around a smelter in southern China reached 75.4 mg kg^−1^^28^. Cd contamination is dangerous, affecting the food chain, and leading to various diseases^1,36,37^. However, rice is a major dietary Cd source for humans because it can efficiently accumulate Cd in grains^38,39^. To control Cd pollution in rice, researchers have focused on studying changes in Cd activity in paddy soil, Cd uptake and transport in rice, and nutrient inhibition of Cd uptake and other aspects^40,41^. It is known that selenium is an important element for living things^42^, and numerous studies have shown that exogenous Se application in rice can increase yield and quality^43–47^, enhancing Se concentrations in the edible parts and reducing Cd transportation and uptake by rice strongly affected by Se bioavailability^14,20,48^. The experimental results confirm that Se application to rice is beneficial for balancing forage and grain harvest through RR use. Therefore, it is completely feasible and necessary to appropriately apply Se fertilizer during the rice growth period solely from the perspective of improving rice yield and quality under feed and grain dual cropping^20^.

Since plant response to Cd toxicity varies with rice cultivars, and so the effect of Se application on Cd accumulation in two rice varieties was investigated^49,50^. The experimental results demonstrated that the effect of Se treatments on Cd concentration varied in different rice tissues^51,52^. In TX rice, Cd accumulation in grains was significantly inhibited in four Se treatments, as well as in the subsequent season stems by the three foliar Se spraying treatments. TX TF_stem/root_ could be reduced by 28.57–39.29%. Compared with the control, TF_leaf/stem_ decreased by 58.54 and 64.71% (P < 0.05). This indicates that moderate application of Se in Cd contaminated paddy soil can effectively inhibit Cd transport from rice roots to aboveground parts and reduce Cd accumulation in grains, thereby alleviating Cd toxicity. Exogenous Se addition significantly reduced Cd concentration (Fig. 1b) and increased Se content in rice tissues (Fig. 2a), which is in agreement with previous studies^5,31,45,53,54^.

Se can increase root NPT (non protein thiol substances) content, which can passivate cell Cd and transport it to vacuoles for storage^55,56^. Se application alters soil Cd bioavailability and reduces its accumulation in rice grown in Cd-contaminated soil^43^. Moreover, the expression of genes related to Cd absorption was decreased at the molecular level^57^. Moderate Se application can inhibit the expression of rice Cd transport genes such as OsIRT1, OsIRT2, OsLCT1, and OsNramp5, thereby reducing Cd upward transport^42^. This can explain the Cd decrease in TF _stems/roots_ in the rice current season. The high Se transport coefficient rebound may be due to excessive Se application by exogenous sources, which strengthens the plant compartmentalization mechanism, which will transport most of the heavy metals absorbed by the roots to the above-ground part for storage, thus driving Cd transport to above-ground part and increasing its content^58^. However, under the combined influence of different seasons and rice varieties, Se application had no significant effect on Cd transport in different subsequent-season rice tissues. After twice foliar spraying Se (T3, at the seedling stage and tillering stage) to the LY variety, Cd content in the current rice straw season was effectively reduced by 18.99%, while in the subsequent rice season it was significantly reduced by 20.00%. However, there was no significant decrease in rice Cd content under other application rates. TX variety experimental results showed a similar trend, and rice Cd content decreased significantly by 46.67% (Fig. 1b).

Generally, moderate Se application to soil or leaves can significantly increase Se content in various rice tissues. Se treatment of the LY variety significantly increased Se content in roots, stems, and leaves by 1.63 times (P < 0.05, Fig. 2a). Moreover, the Cd enrichment coefficient in rice logarithmically decreased with its Se content (Fig. 4), indicating that appropriate application of Se could effectively reduce Cd accumulation in rice. However, the Cd reduction effect in current and subsequent season rice crops differed among the two varieties, which is similar to previous research results^51^. This reflects that there are certain differences in the response of different rice varieties to Se. For example, compared to common rice line Luhui17, D62B was a great potential breeding material with a weaker Cd translocation capacity to shoots^59^. Soil properties and cultivars determine heavy metal accumulation in rice grain and cultivars respond differently to Cd stress^45,59,60^.

Cd absorption capacity of roots of current season rice varieties may be enhanced with selenite application, which may increase root Cd content (Fig. 1), resulting in a poor Cd reduction effect on current season rice. Under the influence of seasonal change and current season root exudates, soil pH and available Cd content in the subsequent season were significantly lower than in current season (Table 2). There was no significant change in the Cd transport coefficient in different rice parts treated with Se application (Fig. 2). However, root Cd content in the subsequent season decreased as a whole and Cd content in all rice parts was significantly reduced (Fig. 1). Se application in Cd-contaminated paddy soil significantly decreased Cd accumulation in the subsequent season by reducing Cd uptake by roots. This is mainly due to the rapid absorption of selenite by soil after application, and the high proportion of HSeO^3−^ morphology under acidic conditions (pH 4.5–5.0)^12^, which is beneficial for crop absorption and thus reducing Cd in subsequent rice.

**Table 2:** Soil pH and available Cd of ratooning rice.

Se treatment			pH	Available Cd mg kg^−1^
LY	Current season	CK	5.34 ± 0.01a	0.22 ± 0.01c
		T1	5.36 ± 0.03a	0.23 ± 0.02b
		T2	5.21 ± 0.02b	0.23 ± 0.02b
		T3	5.24 ± 0.04b	0.24 ± 0.01a
		T4	4.84 ± 0.03c	0.23 ± 0.02b
	Subsequent season	CK	5.30 ± 0.03a	0.39 ± 0.03ab
		T1	5.16 ± 0.05b	0.40 ± 0.05a
		T2	5.13 ± 0.01bc	0.38 ± 0.02b
		T3	5.12 ± 0.06c	0.30 ± 0.05c
		T4	5.31 ± 0.03a	0.31 ± 0.02 c
TX	Current season	CK	5.45 ± 0.03ab	0.25 ± 0.03c
		T1	5.57 ± 0.02a	0.27 ± 0.02b
		T2	5.41 ± 0.04b	0.26 ± 0.04bc
		T3	5.44 ± 0.03b	0.30 ± 0.03a
		T4	5.64 ± 0.01a	0.28 ± 0.02b
	Subsequent season	CK	5.15 ± 0.01a	0.32 ± 0.01d
		T1	5.08 ± 0.04b	0.29 ± 0.04c
		T2	5.09 ± 0.02b	0.31 ± 0.02d
		T3	5.06 ± 0.03c	0.36 ± 0.01b
		T4	5.11 ± 0.05a	0.41 ± 0.05a

In potato plants, the presence of Se can mitigate heavy metal induced toxicity, mainly through Se decreasing Cd and/or As concentrations in different plant tissues, demonstrating the antagonistic effect between Se and these two metals^59^. Notably, there was no significant difference in Cd content in subsequent rice crops under different Se application rates, which can be combined with economic considerations to reduce soil Se application rates.

The experimental results might be closely related with enhanced oxidative stress and mechanical force of the cell walls by Se application^57^. Compared to their controls, Cd content in TX rice roots increased in the current season, and conversely, decreased in the subsequent season, which is consistent with their translocation from roots to stems. Collectively, when combined with the Cd bio-accumulation factor analysis, suitable Se application amounts and times could reduce Cd translocation from roots to stems in Cd-contaminated paddy soil, thus mitigating Cd accumulation in grains, which is also supported by other similar evidence^7,11,20,52^. Hu et al.^53^ demonstrated that Se application decreased the Cd bio-accumulation factor in rice roots and shoots. Furthermore, among the Se treatments, the more effective for reducing grain Cd content were T3 and T4 for LY rice, and T1, T2, T3, and T4 for TX rice.

Rice grown in Cd-contaminated soils poses challenges for food production and safety. Generally, Se fertilizers are used to precisely increase Se contents in the edible parts of crops including rice grains, and to enhance nutritional quality and resistance to abiotic stresses^6,15^. Rice is the staple food for c. 60% of the Chinese population, but approximately two-thirds of the cultivated soil lacks Se^61^. In the experiment, T3 treatment more effectively increased Se content in TX rice roots, stems, leaves and grains, and LY rice leaves and grains. Thus, TX rice responded to Se treatments more than LY rice. Moreover, a significantly lower grain Se content occurred in the T1 treatment (Se application in soil) than the three foliage spraying Se treatments (T2, T3, and T4), indicating that Se foliage spraying greatly contributed to grain Se accumulation, providing an environmentally friendly way to improve Se-enriched rice. A similar result for wheat grain was reported by Galinha et al.^62^. Otherwise, herbage quality harvested in the current season was improved after Se treatments, especially decreasing Cd content in TX rice. T3 treatment obviously improved herbage quality, suggesting that dual harvests on existing land could increase yield and improve quality.

As an efficient rice planting mode, RR production and both grass and grain harvesting are currently widely favored^26^. Under moderate Cd pollution, grass and grain combined with soil Se application in the RR production mode increased Se and reduced Cd in rice, and soil application combined with leaf spraying was more effective.

Soil pH is a key environmental factor, mainly because it can form a suitable soluble environment for soil mineral elements, drive microorganism growth and change community structure. Moreover, taking specific measures can constantly change soil pH and change soil microbial community structure^8,62^.

Overall, in our experiment, Se application affected soil pH and available Cd content, but the effect was insignificant. TX variety soil pH clearly changed, but available soil Cd content changed little (Table 2). During crop growth, the root system continuously absorbs base ions such as K^+^ and releases organic acids into the rhizosphere, leading to soil acidification. Furthermore, nitrogen, phosphorus, and potassium fertilizer application before subsequent rice transplanting lowers soil pH. Generally speaking, soil available Cd content is negatively correlated with soil pH, and the subsequent rice soil pH is significantly reduced, which increases its Cd activity^9^. So, Cadmium bioavailability and accumulation in rice grains is controlled by pH and Ca in paddy soils with a high geological background of transportation and deposition^63^. In our study, soil pH in the subsequent season after Se application increased by 0.09–0.40 units compared with the control, and the corresponding Se application treatment reduced available soil Cd content by 9.38% (P < 0.05). This may be closely related to the impact of Se application on soil microbial communities^64^. Furthermore, Se supply alters the subcellular distribution and Cd chemical forms and the expression of transporter genes involved in Cd uptake and translocation in winter wheat (*Triticum aestivum*)^65^.

After foliar spraying, Se absorbed by the leaves can be transported to other organs, ultimately increasing their Se concentration. However, the key components involved in grain Se accumulation after foliar Se application are still unknown. The Se transportation pathways from leaves to grains generally include: (1) direct transportation via the phloem; and (2) indirect transportation, where Se is transported from leaves to adjacent nodes and internodes, and then upwards to grains, or redistributed through the phloem during grain filling and maturation stages^66^. In our study, grain Se concentration was positively correlated with Se content in roots, individual leaves, ears, and glumes (Fig. 4A), indicating that these multiple parts are involved in Se transport to grains. According to the correlation analysis, grain Se mainly came from leaves (Fig. 5B), indicating that it was directly transported through the phloem.

Additionally, there was a significant negative correlation between current season rice stems and leaves with subsequent season leaves (Fig. 4A), further confirming the importance of leaves as Se sources for grains. In wheat, the nodes, especially the first node below the ear, play a very important role in the transportation of mineral elements such as Zn, Cu, Mn, and Cd. Moreover, accumulation of mineral elements in grains is directly controlled by the transport of mineral elements from the xylem to the phloem^3^. In our study, grain Se concentration was significantly positively correlated with Se transport factors from roots to leaves, indicating that it greatly affects Se accumulation in grains (Fig. 4B). Therefore, the key components and transportation processes involved in grain Se accumulation are leaf Se concentration and Se transfer from root to shoot, which differs from wheat^67,68^.

Given that rice tends to accumulate more Cd and further affects human health through the food chain, Se could mitigate Cd toxicity to rice plants^69^. Presently, 70% of rice in China has low Se content, and there is a large market for promoting Se application to reduce Cd technology. However, moderate Se intake in the human body can improve immunity, while excessive intake can lead to organ damage^44^. When implementing Se application technology to reduce Cd, it will be necessary to comprehensively consider soil Se content and effectiveness, rice variety Se rich characteristics and soil type, and be careful not to exceed the rice Se content safety threshold. The experimental results for TX rice showed that the treatment effects of the current, and subsequent season on rice straw and rice were not the same, but both increased selenium and lowered Cd, especially the rice/stem 3 × spraying TF treatment. Although the same treatment effect on LY rice was not stable, the TF rice/stem combination still showed a decreasing trend. According to the "Selenium Content Requirements for Selenium Rich Agricultural Products" (≤ 1.0 mg kg^−1^, T/HNFX 001-2017), rice straw and rice Se content did not exceed the standard (Fig. 1). Therefore, the effect of increasing selenium and reducing Cd is significantly influenced by soil physicochemical properties and rice varieties, which should be comprehensively considered when making a planting plan. This experiment has shown that people can better produce high-quality rice to meet peoples needs^32,70,71^. From an economic and ecological perspective, among the three common Se application methods in agriculture, seed priming and foliar spraying are convenient and low contribution to environmental pollution, which may expose the ideal ecological environment, benefiting sustainable development^72,73^.

There are differences between pot and field conditions, and so further multi- site and long-term field trials should be carried out for different soil types and rice varieties to verify and determine the appropriate economic and safe Se application dosage.

## Conclusions

The current study demonstrated that the commercially produced rice in Cd-contaminated soil could meet higher yield and better quality rice grains and herbage quality requirements via Se foliar spraying and adding to soil. TX rice benefited more than LY rice from decreasing Cd concentration, and foliar spraying Se more effectively mitigated Cd hazard than single Se addition in soil. Moderate Se application in Cd contaminated paddy soil reduced Cd transport foom rice roots to stems in the current season, and its accumulation and transport in rice paddy varieties differed. Although Cd content in RR straw and grain increased, it was still lower than the national standard food safety limit. In RR, harvesting straw in the current season and rice in the subsequent season is good practice for enriching selenium and reducing Cd. The high Cd current season crop can be used as fodder, while Cd content in the subsequent season crop decreases and Se content is increased. Additionally, low temperature in the subsequent season improved rice quality and greatly enhanced rice cultivation efficiency. Moderate Se application in Cd contaminated paddy soil changed soil pHd, leading to changes in Cd content in subsequent season rice roots and rice, although the main influencing factors need to be further determined. In TX and LY rice varieties, when 1.0 mg kg^−1^ Se was applied to the soil the soil Cd content was 1.33 mg kg^−1^, rice Se content significantly increased but was < 1.0 mg kg^−1^ using the subsequent season harvests of forage and rice, and met national food safety standards. However, the appropriate Se application amount for different varieties requires confirmation through field validation.

## Data Availability

All data generated or analyzed during this study are included in this published article.
